# Activation of the G Protein-Coupled Bile Acid Receptor TGR5 Modulates the HCP5/miR-139-5p/DDIT4 Axis to Antagonize Cervical Cancer Progression

**DOI:** 10.3390/ijms25168932

**Published:** 2024-08-16

**Authors:** Jia Su, Yiqi Zhao, Wei-Dong Chen, Yan-Dong Wang

**Affiliations:** 1 State Key Laboratory of Chemical Resource Engineering, College of Life Science and Technology, Beijing University of Chemical Technology, Beijing 100029, China; sujia20090708@163.com (J.S.); zhaoyq_w@163.com (Y.Z.); 2 Key Laboratory of Receptors-Mediated Gene Regulation and Drug Discovery, School of Basic Medical Science, Inner Mongolia Medical University, Hohhot 010107, China; 3 Key Laboratory of Receptors-Mediated Gene Regulation, School of Basic Medical Sciences, Henan University, Kaifeng 475001, China

**Keywords:** TGR5, LncRNA, HCP5, cervical cancer, miRNA

## Abstract

A growing body of evidence indicates that the G protein-coupled bile acid receptor, TGR5, plays a critical role in multiple physiological processes ranging from metabolic disorders to cancers. However, the biological functions of TGR5 in cervical cancer (CC) have not been elucidated. Here, using *TGR5* knockout mice, we found that a deficiency of TGR5 leads to greater sensitivity to the progression of cervical inflammation. Activation of TGR5 by its specific ligands significantly attenuated the malignant behavior of CC cells. In addition, we found that TGR5 can negatively modulate the expression of lncRNA *HCP5* by blocking its transcription activation when mediated by p65. *HCP5* was highly expressed in CC tissues, which was positively correlated with the poor prognosis of CC patients. *HCP5* knockdown notably restrained CC cell proliferation, colony formation, and migration in vitro, and inhibited tumor growth in vivo. Furthermore, HCP5 can function as the molecular sponge for miR-139-5p to upregulate DNA damage-induced transcript 4 (DDIT4) in CC cells. Murine xenograft studies demonstrated that TGR5 suppressed the tumor formation of CC cells and downregulated *HCP5* and *DDIT4* while increasing *miR-139-5p* in the xenografts. Taken together, these findings, for the first time, indicate that TGR5 inhibits CC progression by regulating the *HCP5*/*miR-139-5p*/DDIT4 axis, suggesting that it may represent a novel and potent target for CC treatment.

## 1. Introduction

Cervical cancer (CC) is one of the most common malignant cancers in women, causing over 34,000 deaths annually all over the world [1]. The high incidence and mortality rate of CC make it a risky threat to women’s health [2]. With the development of clinical treatment strategies such as surgery, radiotherapy, and chemotherapy, the clinical outcome of early-stage cervical cancer has been improved significantly; however, treatment of those with advanced-stage cervical cancer remains unsatisfactory [3]. Therefore, further studies on the molecular mechanisms underlying the occurrence and development of CC remain a top priority, especially those that explore new methods for early diagnosis and treatment.

Takeda G protein-coupled receptor-5 (TGR5), also known as GPBAR1, is a novel transmembrane bile acid (BA) receptor and is involved in multiple pathological processes [4]. Previous studies have shown the critical role of TGR5 in energy homeostasis and glucose metabolism [5]. More than that, increasing evidence demonstrates that TGR5 is closely associated with the inflammatory process and cancer development. For example, activation of TGR5 has been shown to inhibit the inflammatory response by modulating the NLRP3 inflammasome [6]. Our group has implicated TGR5 as a negative regulator in the development of hepatitis and gastritis [7,8]. In addition, TGR5 has been shown to have anti-tumor activity in liver and kidney cancers [9,10]. Interestingly, TGR5 was found to be a tumor promoter in lung cancer [11]. The bidirectional role of TGR5 in different cancers may be related to its tissue specificity. However, to date, the role of TGR5 in the progression of CC and the regulatory mechanisms involved remain unclear.

Long non-coding RNAs (LncRNAs) are a subclass of non-coding RNAs that are longer than 200 nucleotides without the ability to encode proteins. Until now, a mounting number of reports indicated that lncRNAs played a crucial role in multiple cancers, including CC [12,13]. For instance, lncRNA *surfactant associated 1* (*SFTA1P*) is highly expressed in CC tissues and directly interacts with polypyrimidine tract binding protein 1 (PTBP1) to facilitate the proliferation, migration, and invasion progression of CC cells through enhancing the mRNA stability of tropomyosin 4 (TPM4) [14]. LncRNA *LINC00511* functions as an oncogene in CC by promoting the malignant behavior of CC cells through competitively binding to *miR-497-5p* to upregulate the expression of MAPK1 [15]. LncRNA *lymph node metastasis-associated suppressor* (*LNMAS*) is closely associated with lymph node metastasis; its inhibition drives the EMT and immune evasion via enhancing the interaction between high mobility group box 1 (HMGB1) and BRG1, which contributes to the metastatic progression of CC [16]. In sum, lncRNA-based diagnostic and therapeutic targets may greatly benefit from the clinical management of CC.

In this study, we demonstrate that TGR5 reduced the expression of *HCP5*, leading to the induction of *miR-139-5p* and reduction of DNA damage-induced transcript 4 (DDIT4), which attenuates the development of CC both in vivo and in vitro. These findings show that TGR5 exerts an anti-tumor effect by regulating the *HCP5*/*miR-139-5p*/DDIT4 signaling axis, which is a novel anti-tumor mechanism of TGR5, suggesting that the TGR5/*HCP5*/*miR-139-5p*/DDIT4 axis may be a promising target for CC treatment.

## 2. Results

### 2.1. TGR5 Is Downregulated in CC, and Its Deficiency Promotes Cervical Inflammation Response

**Figure 1:**
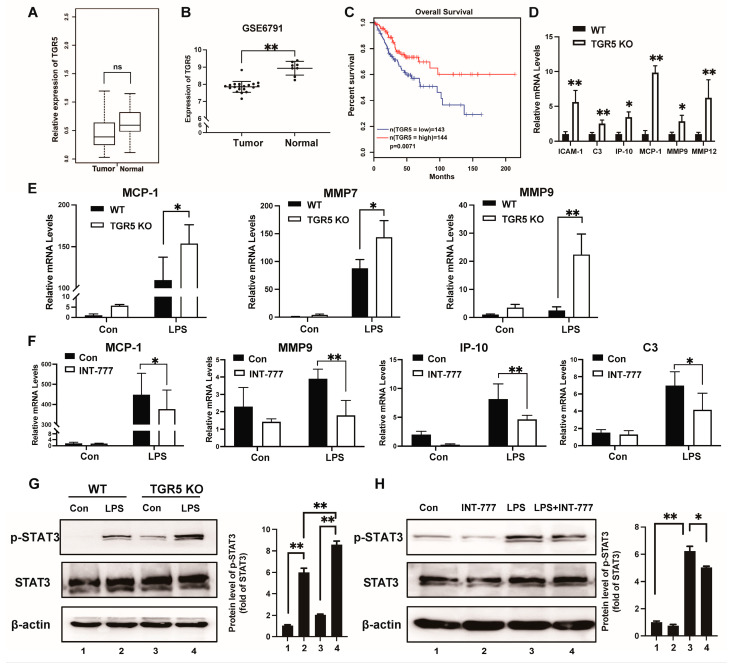
TGR5 is downregulated in CC and its deficiency promotes a cervical inflammation response. (**A**,**B**). TGR5 expression in normal and tumor tissues was analyzed using the TCGA-CESC database and GSE6791 dataset. (**C**). GEPIA analysis of the overall survival of CC patients with high (*n* = 143) TGR5 expression and low *(n* = 144) expression. (**D**). Relative mRNA levels of pro-inflammatory genes in the cervices of WT and TGR5 KO mice (*n* = 5). (**E**). Relative mRNA levels of pro-inflammatory genes in the cervices of WT and TGR5 KO mice after LPS treatment. (**F**). TGR5 ligand treatment suppressed the mRNA levels of pro-inflammatory genes in the cervices of WT mice. (**G**). Western blots show phosphorylated STAT3 (p-STAT3) and total STAT3 (STAT3) in whole proteins from TGR5 KO mice cervices after being treated with LPS for 6 h (*n* = 5). (**H**). Western blots show p-STAT3 and STAT3 in whole proteins from WT mice cervices after being treated with LPS. Mice were fed with a diet containing 20 mg INT-777/kg or a standard rodent diet for three days. After fasting overnight, mice were injected intraperitoneally with LPS for 6 h (*n* = 5). WT, wild-type mice; TGR5 KO, TGR5 knockout mice. * *p* < 0.05, ** *p* < 0.01, ns means not significant.

The expression levels of TGR5 were investigated using The Cancer Genome Atlas (TCGA) database (http://cancergenome.nih.gov/, accessed on 1 July 2020). We found that TGR5 expression was at a lower level in cervical cancer than in non-tumor tissues (Figure 1A). Consistent with the results in the TCGA database, the expression of TGR5 was downregulated in cancer tissues according to the GSE6791 dataset (Figure 1B). Meanwhile, overall survival (OS) analysis showed that patients with low TGR5 levels had shorter survival times than patients with high TGR5 levels (Figure 1C). Taken together, these results verify that TGR5 might act as a negative regulator during CC progression. 

Next, we compared the expression of proinflammatory genes in the cervices of both *TGR5* knockout (KO) and WT mice. We found that intercellular adhesion molecule 1 (*ICAM1*), complement C3 (*C3*), interferon-inducible protein (*IP*)-10, chemokine C-C motif ligand 2 (*MCP-1*), matrix metallopeptidase 9 (*MMP9*), and matrix metallopeptidase 12 (*MMP12*) were differentially upregulated in TGR5 KO mice (Figure 1D). We then compared the mRNA levels of proinflammation genes in the cervices of both TGR5 KO and WT mice after being treated with lipopolysaccharide (LPS). The upregulation of *MCP-1*, *MMP7*, and *MMP9* induced by LPS was more intense in TGR5 KO mice than in WT mice (Figure 1E). Furthermore, we tested whether ligand-activated TGR5 could downregulate the expression levels of these proinflammatory genes induced by LPS. INT-777 treatment, which can activate TGR5, suppressed LPS-induced *MCP-1*, *MMP9*, *IP-10*, and *C3* expression in the cervix tissues of WT mice (Figure 1F). Persistent activation of STAT3 has been found in various cancer types, including cervical cancer. It is regarded as a crucial regulator during the initiation and progression of cancers [17,18]. Furthermore, STAT3 signaling has been demonstrated to promote inflammation-regulated carcinogenesis [19]. We observed that the phosphorylation of STAT3 in TGR5 KO cervices was much higher than that in WT cervices under the LPS treatment (Figure 1G). Then, we used LPS to enhance the activation of the STAT3 pathway in the mouse cervix. TGR5 agonist treatment significantly weakens LPS-induced STAT3 phosphorylation in WT mice (Figure 1H). These results show that TGR5 acted as a negative modulator in the cervical inflammation response and was closely related to CC progression.

### 2.2. TGR5 Activation Suppresses Cervical Cancer Progression through the Downregulation of HCP5 Expression

**Figure 2:**
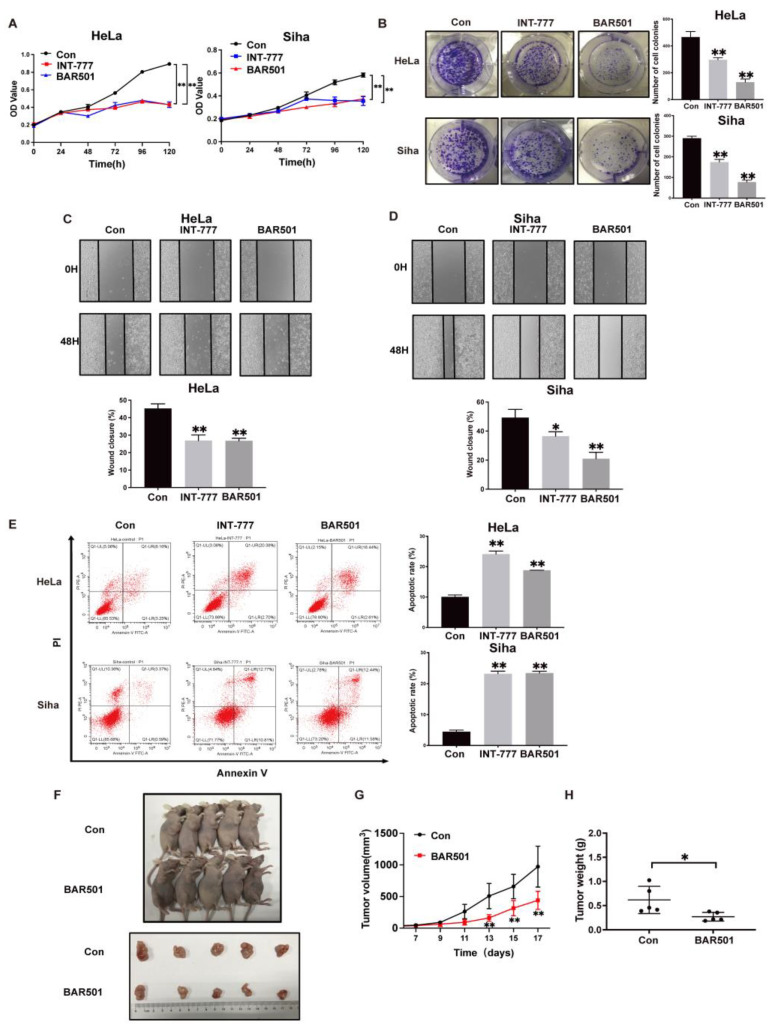
TGR5 activation suppresses cervical cancer progression both in vivo and in vitro. (**A**). TGR5 activation by its ligand inhibits the proliferation of HeLa and Siha cells. (**B**). TGR5 ligand treatment suppressed the CC cell colony formation. (**C**,**D**). Activation of TGR5 in HeLa and Siha cells exhibited a lower scratch closure rate than the controls. (**E**). Flow cytometry analysis indicated that TGR5 ligands promoted the cell apoptosis process. (**F**). Four-week-old female nude mice were subcutaneously inoculated with HeLa cells followed by treatment with the vehicle or BAR501 20 mg/kg through intraperitoneal injection. The mice were sacrificed and photographed (*n* = 5). (**G**). The tumor volumes were measured every two days during the treatment. (**H**) Tumor weights were also recorded. * *p* < 0.05, ** *p* < 0.01.

To evaluate the function of TGR5 in cervical cancer, we first activated TGR5 with its ligands, INT-777 and BAR501, to test the effect of TGR5 activation on CC cell growth and migration. As shown in the MTT assay, TGR5 ligand treatment significantly suppressed the cell growth of HeLa and Siha cells (Figure 2A). Similarly, colony-forming assays revealed that TGR5 activation impaired the cell colony growth rate (Figure 2B). A wound-healing assay further demonstrated that cells treated with INT-777 or BAR501 exhibited a lower wound closure rate than the control group (Figure 2C,D). Next, flow cytometry was performed to test whether TGR5 activation could affect CC cell apoptosis. As shown in Figure 2E, the ratio of apoptotic cells was significantly increased after being treated with INT-777 or BAR501 compared with the untreated group. To further determine the antitumor role of TGR5 in vivo, we subcutaneously injected HeLa cells into female nude mice to establish a xenograft model. Once the average tumor volume reached 100 mm^3^, mice were treated with BAR501 or the vehicle through intraperitoneal injection. During the treatment period, the size and weight of tumors in the BAR501-treated group were significantly smaller and lighter than those in the control mice (Figure 2F–H). Overall, these results suggested that the activation of TGR5 inhibited cervical cancer cell growth and migration in vitro, and impaired tumor growth in vivo.

**Figure 3:**
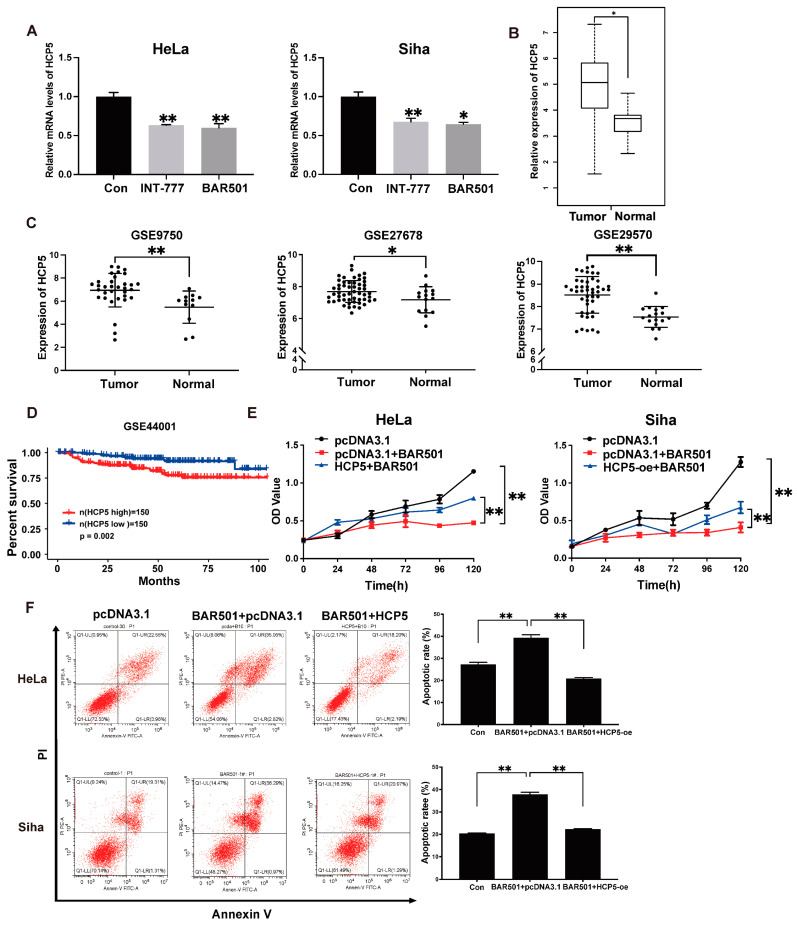
TGR5 activation antagonizes CC through downregulating *HCP5* expression**.** (**A**). *HCP5* expression levels in HeLa and Siha cells were inhibited after being treated with TGR5 for 48 h using qRT-PCR. (**B**). The expression levels of *HCP5* in CC tissues and normal tissues from the TCGA database. (**C**). GSE9750, GSE27678, and GSE29570 datasets suggested that *HCP5* was highly expressed in CC tissues compared to the non-tumor ones. (**D**). Survival curve of the analysis of the overall survival of CC patients with high (*n* = 150) and low (*n* = 150) *HCP5* expression, based on the GSE44001 dataset. (**E**). The MTT assay showed the effect of *HCP5* overexpression, combined with the TGR5 ligand, on cell proliferation. (**F**). The effect of *HCP5* overexpression combined with BAR501 on CC cell apoptosis, determined by flow cytometry analysis. * *p* < 0.05, ** *p* < 0.01.

Next, to elucidate the related lncRNAs involved in the anti-tumor effect mediated by TGR5 activation, we performed the lncRNA microarray to evaluate the dysregulated lncRNAs in response to TGR5 activation in HeLa cells. Among these differentially expressed lncRNAs, lncRNA *HCP5* drew our attention (Table S3). The downregulation of *HCP5* under TGR5 ligand treatment was first confirmed in HeLa and Siha cells through qRT-PCR (Figure 3A). Subsequently, we analyzed the expression of *HCP5* in CC and adjacent tissues in the TCGA database and found that *HCP5* was highly expressed in CC tissues (Figure 3B). Consistent with the results in TCGA, the overexpression of *HCP5* was further confirmed in another three CC datasets (GSE9750, GSE29570, and GSE27678), providing further evidence of its aberrant upregulation in CC (Figure 3C). According to the result of the Kaplan–Meier survival analyses, high expression of *HCP5* was found to be significantly associated with lower overall survival, indicating its prognostic value in CC (Figure 3D). Therefore, we turned our attention to *HCP5* for further investigation. 

To evaluate the potential function of *HCP5* in the TGR5 activation-induced anti-tumor effect, we upregulated the *HCP5* expression in CC cells using the *HCP5* overexpression plasmid. The MTT assay showed that the cell growth inhibition induced by TGR5 activation was significantly attenuated by overexpressing *HCP5* in HeLa and Siha cells (Figure 3E). Furthermore, the increase in apoptotic cell numbers due to the activation of TGR5 was restored by *HCP5* in CC cell lines (Figure 3F). These results indicated that TGR5 impaired CC progression through regulating *HCP5*.

**Figure 4:**
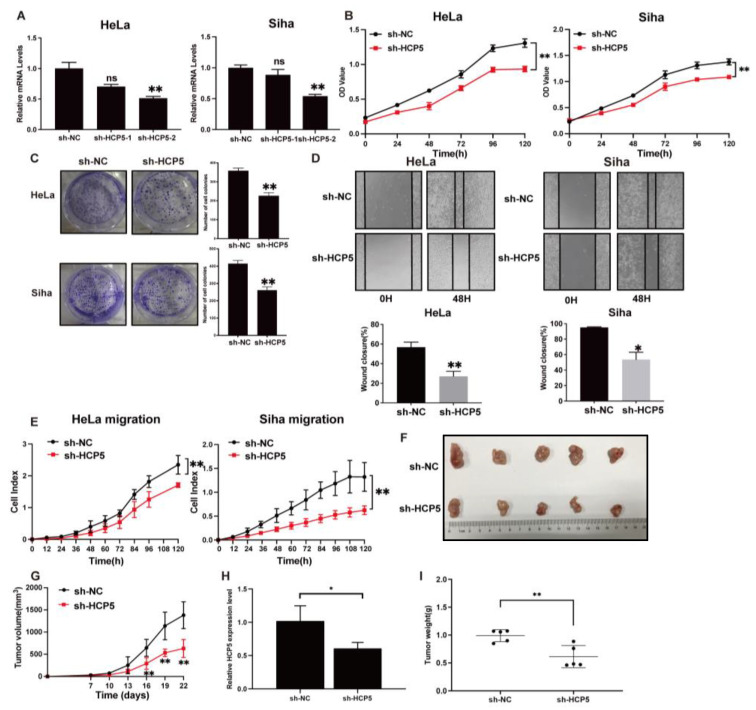
*HCP5* drives CC progression in vitro and in vivo. (**A**). The qRT-PCR analysis of *HCP5* in HeLa and Siha cells post-transfected with sh-NC or sh-HCP5. (**B**,**C**). MTT and cell colony formation assays were conducted, respectively, to assess the effect of *HCP5* inhibition on cell proliferation. (**D**,**E**). The migratory ability of CC cells with *HCP5* knockdown was analyzed using a wound-healing assay and RTCA. (**F**–**I**). Tumor xenograft experiments were done to examine the impact of *HCP5* on tumor growth. Tumor images were photographed, and tumor growth curves and weights were also recorded. * *p* < 0.05, ** *p* < 0.01, ns means not significant.

To further elucidate the role of *HCP5* in CC, short hairpin RNAs (shRNAs) were used to knock down *HCP5* in HeLa and Siha cells specifically. The knocking-down efficiency was determined using qRT-PCR (Figure 4A). MTT and cell colony formation assays indicated that the knockdown of *HCP5* significantly inhibited cell growth and the proliferation of HeLa and Siha cells (Figure 4B,C). A wound-healing assay was performed to test the effect of *HCP5* on the migration of CC cell lines. The results showed that the knockdown of *HCP5* in HeLa and Siha cells significantly impaired the migration distance (Figure 4D). Additionally, we performed the cell migration assay using the xCELLigence^®^ RTCA DP instrument system. As expected, the knockdown of *HCP5* by shRNA suppressed cell migration (Figure 4E). To further verify the effect of *HCP5* on tumor growth in vivo, HeLa cells transfected with sh-HCP5 or sh-NC were subcutaneously injected into nude mice to establish a xenograft model. As shown in Figure 4F, tumors formed in the sh-HCP5 group were significantly smaller than those in the control group. Compared to the control group, the efficient knockdown of *HCP5* resulted in a significant reduction in tumor volume and weight (Figure 4G–I). Collectively, these findings suggested that *HCP5* enhances CC cell proliferation and migration, and promotes tumor growth in vivo, thus contributing to CC progression. 

### 2.3. TGR5 Suppressed HCP5 Expression Level through Transcriptional Inactivation

**Figure 5:**
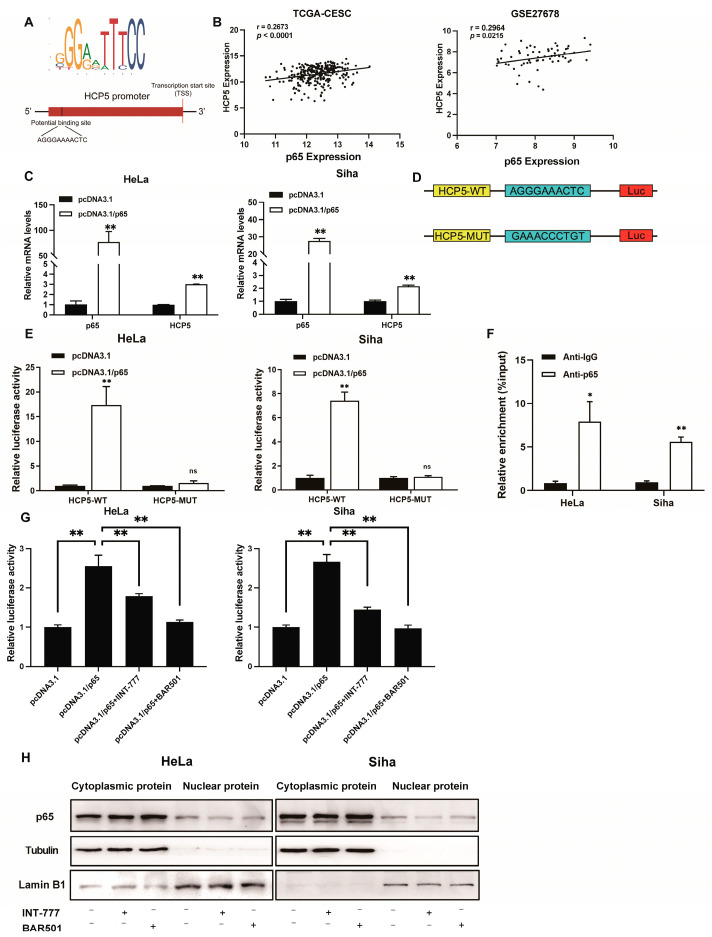
TGR5 suppressed *HCP5* expression level through transcriptional inactivation. (**A**). The potential binding sites of p65 predicted by JASPAR. (**B**). Correlation analysis between *HCP5* expre-ssion and p65 expression in CC tissues based on TCGA data and the GSE27678 dataset. (**C**). The qRT-PCR was performed to examine the relative expression of *HCP5* in CC cells transfected with p65 overexpression vectors. (**D**). The luciferase vector of the *HCP5* promoter region, containing potential p65 binding sites, was simulated. (**E**). The luciferase vector carrying the *HCP5* promoter region was co-transfected with p65 plasmid or control plasmid into HeLa and Siha cells. (**F**). The binding sites in which p65 binds to the *HCP5* promoter were determined using a ChIP assay. (**G**). The impact of TGR5 activation on the p65-induced upregulation of *HCP5* transcriptional activity was confirmed through a luciferase assay. (**H**). The translocation of p65 in HeLa and Siha cells after TGR5 activation was measured through a western blot assay. * *p* < 0.05, ** *p* < 0.01, ns means not significant.

Next, we sought to uncover the mechanisms by which TGR5 modulates *HCP5* expression in CC cells. Previous studies have shown that transcriptional factors (TFs) can modulate the expression level of some lncRNAs at the transcriptional level [20,21]. By using the UCSC and JASPR analysis tools, we found a putative p65 binding site in the promoter region of *HCP5* (Figure 5A). Furthermore, we conducted the correlation analysis between p65 and *HCP5* based on the TCGA and the GSE27268 datasets. As shown in Figure 5B, p65 expression was positively correlated with *HCP5* expression in cancer tissues. Thus, we hypothesized that p65 might function as the transcriptional activator of *HCP5*. To confirm this hypothesis, we detected the *HCP5* expression after overexpressing p65 in CC cells, and qRT-PCR results showed that p65 significantly upregulated the expression of *HCP5* (Figure 5C), which indicated that p65 was essential for the upregulation of *HCP5* in CC. To determine the interaction between p65 and the *HCP5* promoter, we performed luciferase reporter experiments to clarify their modulation mechanism. As shown in Figure 5D, promoter–reporter plasmids with or without putative binding sites of p65 were co-transfected with p65 plasmids into CC cells. The luciferase activity of pGL4.1/HCP5-WT was increased upon p65 overexpression, and that of pGL4.1/HCP5-MUT hardly changed under the same treatment (Figure 5E). Furthermore, a Chromatin immunoprecipitation assay (ChIP) was performed to prove the interaction between p65 and the *HCP5* promoter. The ChIP assay results showed that the fragment of the *HCP5* promoter was significantly enriched by the anti-p65 antibody compared to the negative control, suggesting that p65 binds to the promoter region of *HCP5* (Figure 5F). Thus, our findings proved that p65 could enhance the transcription of *HCP5* in CC cells. Next, we hypothesized whether TGR5 activation could block the p65 transcriptional induction of *HCP5*, resulting in *HCP5* downregulation. As shown in Figure 5G, the upregulation of pGL4.1/HCP5-WT vector activities induced by p65 was abolished after treatment with TGR5 ligands in HeLa and Siha cells. As we all know, p65 drives its target genes’ transcription level after translocating into the nucleus. Thus, we conducted a western blot to detect the p65 protein in nuclear/cytoplasmic extraction from CC cells after being treated with TGR5 ligands. As shown in Figure 5H, the protein level of p65 in the nucleus was downregulated once TGR5 activated, which indicated that TGR5 could inhibit the translocation of p65 in CC cell lines. Taken together, these findings demonstrate that TGR5 could negatively regulate *HCP5* expression through attenuating p65 binding to the promoter region of *HCP5*, contributing to *HCP5* downregulation. 

### 2.4. HCP5 Functions as a Sponge to miR-139-5p to Target DDIT4 in CC Cells

**Figure 6:**
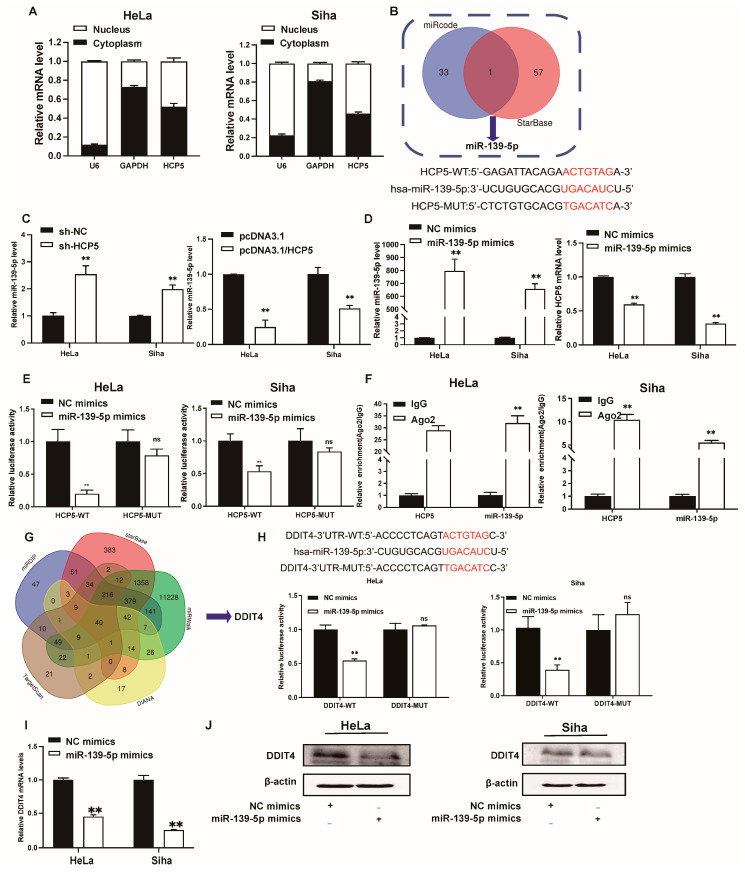
*HCP5* functions as a sponge to *miR-139-5p*, targeting *DDIT4* in CC cells. (**A**). The subcellular location of *HCP5* in CC cells was analyzed through a qRT-PCR, and *U6* and *GAPDH* were set as the cytoplasmic and nuclear control, respectively. (**B**). The potential *miR-139-5p* binding sites in *HCP5*. (**C**). Relative expression of *miR-139-5p* in CC cells after *HCP5* inhibition and overexpression was examined through a qRT-PCR. (**D**). The qRT-PCR analysis of *miR-139-5p* and *HCP5* expression in HeLa and Siha cells after overexpression of *miR-139-5p*. (**E**). The dual-luciferase assays were performed to verify the binding sites between *miR-139-5p* and *HCP5* in CC cells. (**F**). Analysis of *miR-139-5p* and *HCP5* via qRT-PCR in anti-AGO2 or IgG immunoprecipitated RNA. (**G**). Venn diagram of the target gene of *miR-139-5p* using online tools Starbase, miRWalk, DIANA, TargetScan, and miRDIP. (**H**). The binding sites between *miR-139-5p* and *DDIT4* were verified by dual-luciferase reporter assay. (**I**,**J**). The mRNA levels and protein levels of DDIT4 in HeLa and Siha cells after transfection with negative control (NC) mimics or *miR-139-5p* mimics were analyzed via qRT-PCR and western blot, respectively. ** *p* < 0.01, ns means not significant.

Recently, mounting evidence has proved that lncRNAs may function as competing endogenous RNAs (ceRNAs) to interact with specific miRNAs, which are involved in modulating tumor progression [22,23]. As shown in Figure 6A, *HCP5* has been distributed in the cytoplasm and nucleus of CC cells, demonstrating that it may modulate target expression at the posttranscriptional level. By using two different online bioinformatic pieces of software (Starbase, http://starbase.sysu.edu.cn/ and miRcode, http://www.mircode.org/, accessed on 5 January 2021), a potential binding site of *miR-139-5p* has been found in the *HCP5* sequence (Figure 6B). We also found that *miR-139-5p* was significantly downregulated in CC tissues from the TCGA-CESC dataset (Figure S1A, accessed on 15 January 2021). Thus, *miR-139-5p* was selected for further investigations. The results of the qRT-PCR indicated that the knockdown of *HCP5* in CC cells significantly increased *miR-139-5p* levels.

In contrast, *HCP5* overexpression exerted the opposite effect (Figure 6C). Additionally, *HCP5* was significantly repressed after transfecting *miR-139-5p* mimics into CC cells (Figure 6D). To clarify further whether *HCP5* acts as a molecular sponge to *miR-139-5p* in CC, a dual-luciferase reporter assay was performed, and the results showed that the luciferase activity of HCP5-WT was decreased when it was co-transfected with *miR-139-5p* mimics, while the inhibitory effect induced by *miR-139-5p* was abolished in HCP5-MUT plasmids (Figure 6E). To further elucidate the regulatory mechanism between *miR-139-5p* and *HCP5*, we performed the RNA-binding protein immunoprecipitation (RIP) assays. We found that both *miR-139-5p* and *HCP5* were significantly enriched in the AGO2 group compared to the IgG group in both HeLa and Siha cells (Figure 6F). To further illuminate the biological role of *miR-139-5p* in CC, MTT assays and wound-healing assays were performed. The results showed that the proliferation and migration of HeLa and Siha cells were significantly inhibited after they were transfected with *miR-139-5p* mimics (Figure S1B,C). 

Next, to elucidate the target gene of *miR-139-5p*, we used five online miRNA target-predicting tools (Starbase, miRWalk, DIANA, TargetScan, and miRDIP) to analyze its potential downstream targets. We found 40 candidate genes (Figure 6G). Among them, DDIT4 was found to be elevated in CC tissues from the TCGA-CESC dataset and was positively associated with the poor overall survival of CC patients (Figure S2A,B). In addition, the correlation analysis showed that DDIT4 positively correlated with lncRNA *HCP5* in CC tissues (Figure S2C). Therefore, *DDIT4* was identified as the target gene of *miR-139-5p* for further studies. We then cloned a fragment of the *DDIT4* 3′UTR, which contained the predicted *miR-139-5p* binding site (*DDIT4*-WT) or the mutant binding site (*DDIT4*-MUT), into luciferase reporter plasmids, and subsequently co-transfected these vectors with *miR-139-5p* mimics into HeLa and Siha cells (Figure 6H). Luciferase assays revealed that *miR-139-5p* notably reduced the luciferase activity of the DDIT4-WT plasmid but did not affect the DDIT4-MUT plasmid (Figure 6H). Furthermore, both the mRNA and protein levels of DDIT4 were notably decreased in HeLa and Siha cells after they were transfected with *miR-139-5p* mimics (Figure 6I,J). To further explore the biological function of DDIT4 in CC, we knocked down its expression in CC cells through transfection with siRNA. The knockdown efficiency was confirmed using a qRT-PCR (Figure S2D). The MTT assays and scratch assays revealed that the knockdown of *DDIT4* inhibited cell proliferation and migration (Figure S2E,F). In addition, *DDIT4* downregulation induced apoptosis in CC cell lines (Figure S2G). Taken together, these data suggest that *HCP5* regulates the expression of *DDIT4* through competitively sponging *miR-139-5p* in CC.

### 2.5. Activation of TGR5 Inhibits CC Progression by HCP5, Which Promotes CC Progression by Modulating the miR-139-5p/DDIT4 Axis In Vivo and In Vitro

**Figure 7:**
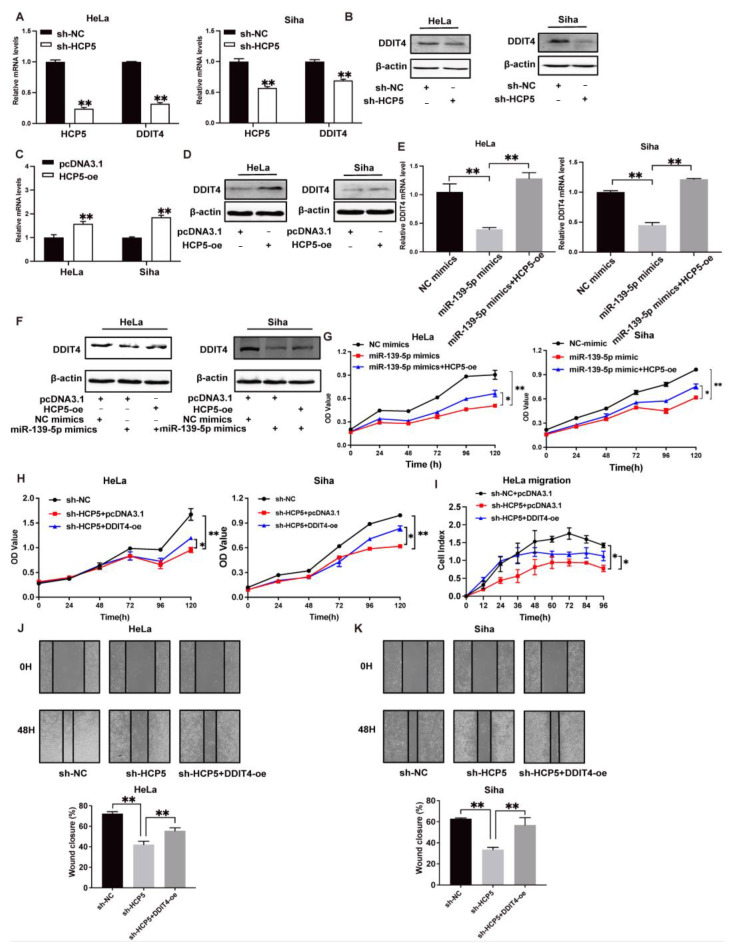
*HCP5* promotes CC progression by modulating the *miR-139-5p/DDIT4* axis. (**A**). *DDIT4* mRNA levels in CC cells after *HCP5* inhibition were examined via qRT-PCR. (**B**). Western blot was conducted to analyze DDIT4 protein levels in HeLa and Siha cells after transfection with shRNA (sh-NC) or shHCP5. (**C**,**D**). The mRNA and protein levels of DDIT4 in CC cells with *HCP5* overexpression were analyzed via qRT-PCR or western blot. (**E**,**F**). The mRNA and protein levels of DDIT4 in *HCP5* overexpression HeLa and Siha cells transfected with negative control (NC) mimics or *miR-139-5p* mimics were detected via qRT-PCR and western bot. (**G**,**H**). The MTT assay in HeLa and Siha with co-transfection of *miR-139-5p* mimic and pcDNA3.1/HCP5 or shHCP5 and pcDNA3.1/DDIT4. (**I**–**K**). The migratory ability of HeLa and Siha cells co-transfected with shHCP5 and pcDNA3.1/DDIT4 were examined via RTCA and cell scratch assay. * *p* < 0.05, ** *p* < 0.01.

Subsequently, we further explored whether *HCP5* exported its role in CC cells by modulating the *miR-139-5p*/*DDIT4* axis. Both the mRNA and protein levels of DDIT4 were significantly reduced in HCP5-silenced CC cell lines (Figure 7A,B), whereas ectopic *HCP5* expression displayed the opposite effect (Figure 7C,D). To verify the role of *miR-139-5p* in the regulatory mechanism between *HCP5* and DDIT4, we performed the rescue experiment through co-transfecting it with the *HCP5* plasmid and *miR-139-5p* mimics into HeLa and Siha cells, and subsequently examined mRNA and protein levels using qRT-PCR and immunoblotting, respectively. As shown in Figure 7E,F, both the mRNA and protein levels were significantly suppressed by *miR-139-5p*, and *HCP5* overexpression reversed this inhibitory effect (Figure 7E,F). Furthermore, MTT assays demonstrated that the suppression of cell proliferation induced by *miR-139-5p* in HeLa and Siha cells was partly restored by *HCP5* overexpression (Figure 7G). Additionally, results from the cell proliferation assay indicated that cell growth inhibition caused by *HCP5* silencing was effectively reversed by the ectopic *DDIT4* expression (Figure 7H). In line with the results from the MTT assay, *DDIT4* overexpression partly restored the migration and wound-healing of HeLa and Siha cells impaired by the knockdown of *HCP5* (Figure 7I–K). In summary, these findings indicate that *HCP5* promotes CC progression via modulating the *miR-139-5p/DDIT4* axis.

**Figure 8:**
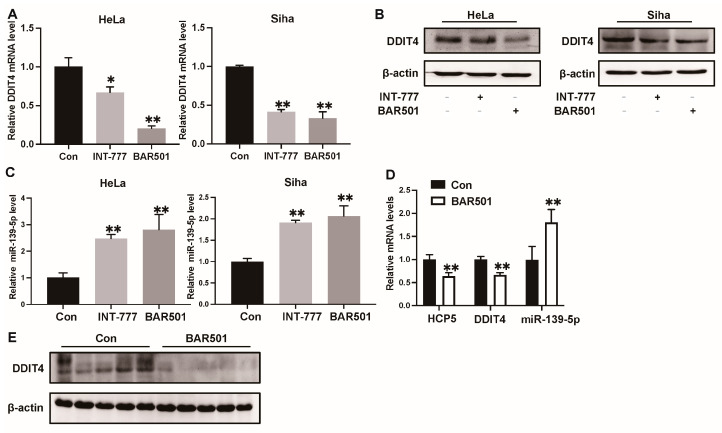
Activation of TGR5 inhibits CC progression by the *HCP5*/*miR-139-5p*/*DDIT4* axis in vivo and in vitro. (**A**,**B**). HeLa and Siha cells were treated with control DMSO, TGR5 ligands INT-777, and BAR501 for 48 h. Then, the expression level of DDIT4 was quantified through qRT-PCR and western blot. (**C**). Relative *miR-139-5p* expression after TGR5 activation. (**D**). The expression of *HCP5*, *miR-139-5p*, and *DDIT4* in the xenografts was detected using qRT-PCR. (**E**). The protein levels of DDIT4 in the xenografts were analyzed via western blot. * *p* < 0.05, ** *p* < 0.01.

**Figure 9:**
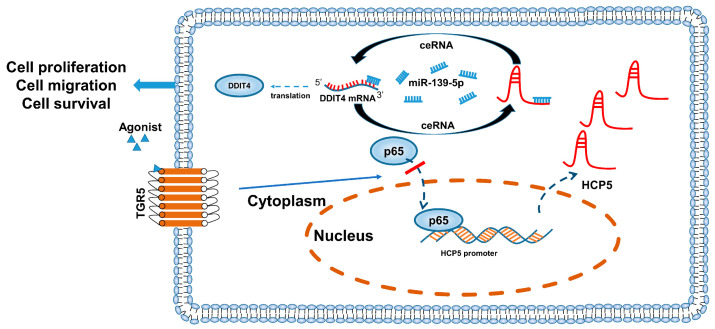
Schematic representation of the role of TGR5 as a tumor suppressor in the progression of CC. Red bar means inhibit. Arrows means move.

These studies have demonstrated that TGR5 can downregulate the expression of *HCP5*, while *HCP5* can competitively sponge *miR-139-5p* to increase the *DDIT4* level. Therefore, we further investigated the effect of TGR5 in the *HCP5*/*miR-139-5p*/*DDIT4* axis. According to the results of the qRT-PCR and western blot assays, activation of TGR5 through different specific ligands significantly reduced the mRNA and protein levels of DDIT4 in CC cell lines (Figure 8A,B). At the same time, it increased *miR-139-5p* expression (Figure 8C). Additionally, we analyzed the expression levels of *HCP5*, *miR-139-5p*, and *DDIT4* in CC xenografts treated with the TGR5 ligand BAR501. The results of the qRT-PCR indicated that TGR5 activation significantly inhibited *HCP5* gene expression and DDIT4 gene and protein expression (Figure 8D,E), while inducing *miR-139-5p* expression (Figure 8D). Based on these findings, we can conclude that the anti-tumor effect of TGR5 activation in the CC xenograft was partially achieved via modulating the *HCP5*/*miR-139-5p/DDIT4* axis in vivo (Figure 9).

## 3. Discussion

TGR5, belonging to the G protein-coupled receptor (GPCR) family, has been found to act as a bile acid membrane receptor [24]. Recently, a mounting number of studies have focused on the role of TGR5 in modulating energy, bile acid, and glucose metabolism, while its role and regulatory mechanism in human CC remained uncovered. In this study, we systematically investigated the biological function of TGR5 in CC, as well as the underlying molecular mechanisms in vivo and in vitro. We demonstrated that TGR5 is downregulated in CC tissues, and its expression is significantly related to a better clinical prognosis. Subsequently, TGR5 greatly inhibits cell proliferation and migration, while inducing cell apoptosis in CC cell lines. In addition, in vivo experiments showed that the activation of TGR5 suppresses tumor growth in mice. 

In the past decade, an increasing number of studies have revealed the crucial role of lncRNAs in the initiation and development of multiple cancers [25,26,27,28]. In cervical cancer, a mounting number of lncRNAs, such as *LINC00511* [29] and *UCA1* [30], have been characterized, and their regulatory mechanisms have been well defined. lncRNA *HCP5* has been reported to play crucial roles in various types of cancer. Recently, Li et al. reported that *HCP5* promotes the proliferation and migration of CC cells through the *miR-216a-5p*/*CDC42* axis [31]. Furthermore, *HCP5* can sponge *miR-15a* to upregulate MACC1, thus promoting the malignant behavior of CC [32]. In this study, we observed that *HCP5* was significantly downregulated once TGR5 was activated in CC. In addition, overexpressed *HCP5* reversed the anti-tumor effect of TGR5 in CC. Subsequently, we further explored the regulatory mechanism between TGR5 and *HCP5*. Firstly, we identified that p65 acted as the transcription factor of *HCP5* and could drive its transcription level. Our previous study revealed that TGR5 can inhibit IκBα phosphorylation and then the nuclear translocation of p65 through enhancing β-arrestin2 interaction with IκBα, resulting in antagonizing the NF-κB signaling pathway in liver cancer cells [7]. In this work, we showed that TGR5 activation inhibited the nuclear translocation of p65 in ovarian cancer cells. We further confirmed that TGR5 repressed *HCP5* expression by reducing the binding of p65 to the *HCP5* promoter region. Thus, it is speculated that TGR5 may enhance the interaction of β-arrestin2 and IκBα to antagonize the nuclear translocation of p65, inhibiting *HCP5* expression. It will be interesting to study the mechanism of TGR5 regulating *HCP5* in future work. In addition, to the best of our knowledge, our work provides the first evidence demonstrating that TGR5 plays a role in modulating lncRNA expression. Therefore, more efforts should be put into the elucidation of other potential lncRNAs which are regulated by TGR5. 

lncRNAs can exert different biological functions to regulate the expression of downstream target genes at transcription, translation, and post-translational modification [33,34]. According to the nucleoplasmic distribution of *HCP5* in CC cell lines, we investigated the downstream regulatory mechanism of *HCP5* in CC. Previous researchers have reported that *HCP5* can act as a molecular sponge to miRNA in different cancers [35,36]. In this study, the luciferase assay and RIP assays indicated that *HCP5* acted as a sponge to *miR-139-5p*. Overexpression of *miR-139-5p* significantly suppressed the proliferation and migration of CC cells. Furthermore, rescue experiments demonstrated that *HCP5* overexpression could reverse the growth-inhibiting effect induced by *miR-139-5p*, which indicates that *HCP5* can sponge *miR-139-5p* to facilitate CC progression. 

We further explored the downstream target genes of *HCP5*/*miR-139-5p* and revealed that DDIT4 is a novel target of *miR-139-5p*. DDIT4 is widely expressed in various tissues and is closely related to cellular stresses, such as hypoxia, ionizing radiation (IR), heat shock, etc. [37]. Previous studies have reported that DDIT4 facilitates the proliferation, migration, and drug resistance of several cancers, including glioma [38], gastric cancer [39], and lung cancer [40]. In this study, DDIT4 was elevated and positively related to *HCP5* expression in CC tissues, while higher DDIT4 expression predicted a worse clinical prognosis. The knockdown of DDIT4 inhibited cell proliferation and migration, while inducing apoptosis in CC cell lines. In addition to this, TGR5 downregulated DDIT4 by reducing *HCP5* in CC cells, while *HCP5* can enhance DDIT4 expression through sponging *miR-139-5p*. In sum, these four molecules form the TGR5/*HCP5*/*miR-139-5p*/*DDIT4* signal axis, which turns out to be a novel anti-CC mechanism of TGR5.

## 4. Materials and Methods

### 4.1. Cell Lines and Cell Culture

The CC cell lines HeLa and Siha were purchased from the Institute of Basic Medical Sciences of the Chinese Academy of Medical Sciences (Beijing, China). The HeLa and Siha cells were cultured in Roswell Park Memorial Institute (RPMI) 1640 culture medium and the Minimum Essential Medium (MEM) culture medium, respectively. All of the culture mediums were supplemented with 10% fetal bovine serum (FBS) (Santa Cruz, CA, USA) and 1% antibiotics (100 U/mL penicillin and 100 ug /mL streptomycin sulfates) (Caisson Laboratories, Smithfield, UT, USA). These CC cells were all maintained in a humidified atmosphere at 37 °C and with 5% CO_2_ in the air. To activate TGR5 in CC cells, the specific TGR5 agonist INT-777 (100 μM, MedChemExpress, Monmouth Junction, NJ, USA) or BAR501 (10 μM, MedChemExpress) was used to treat cells for 48 h. 

### 4.2. Plasmid Construction and Cell Transfection

The full-length lncRNA *HCP5* was cloned into the pcDNA3.1 vector (Invitrogen, Carlsbad, CA, USA) to generate the *HCP5* overexpression plasmid. The p65 and DDIT4 overexpression plasmids were established by inserting the full-length coding sequence (CDS) of p65 and DDIT4 into the pcDNA3.1 vector. The shRNA targeting *HCP5* was purchased from GenePharma (Shanghai, China). The negative control, DDIT4 siRNAs, *miR-139-5p* mimic, and the *miR-139-5p* inhibitor were all acquired from RiboBio (Guangzhou, China). Overexpression plasmids, shRNA targeting *HCP5* (sh-HCP5), siRNAs targeting DDIT4 (si-DDIT4), *miR-139-5p* mimics, and NC mimics, were transfected into CC cells using Lipofectamine 3000 reagent (Invitrogen, Carlsbad, CA, USA). The following experiments were performed 48 h after transfection. The sequences of shRNA and siRNA, and the primers used to construct the overexpression plasmids are shown in Table S1.

### 4.3. RNA Isolation, Reverse Transcription and qRT-PCR

Total RNA from CC cells and xenografts was isolated using TRIZOL reagent (Invitrogen, Carlsbad, CA, USA), following the manufacturers’ directions. For mRNA and lncRNA detection, RNA was reverse transcribed into complementary DNA (cDNA) using the Strand cDNA Synthesis kit (Invitrogen, Carlsbad, CA, USA). For miRNA analysis, reverse transcription was performed using the miRNA First-Strand cDNA Kit (TIANGEN, Beijing, China). The qRT-PCR analysis was carried out using the Power SYBR Green PCR Master Mix protocol (Applied Biosystems, Foster, CA, USA). For the internal control, β-actin and U6 were chosen. The comparative CT (2^−ΔΔCT^) method calculated the relative RNA expression level. The primers for the qRT-PCR are presented in Table S2. 

### 4.4. Subcellular Fractionation

Nuclear and cytoplasmic extractions of CC cells were carried out using the PARIS kit (Life Technologies, Carlsbad, CA, USA), following the directions of the manufacturer. The relative expression of *HCP5*, *GAPDH*, and *U6* in both extractions was determined using a qRT-PCR. 

### 4.5. Cell Proliferation Assay

To analyze the effect of TGR5 activation on cell viability, CC cells were seeded in 96-well plates. Twenty-four hours later, the cells were treated with or without TGR5 ligands, followed by the addition of 10 μL 3-(4,5)-dimethylthiahiazo (-z-y1)-3,5-di- phenytetrazoliumromide (MTT) (Lablead, Beijing, China) in each well. After 4 h of incubation, the purple crystals were extracted using DMSO, and optical density (OD) was measured at 570 nm with a microplate reader. The proliferation curve was determined by the OD value. As for investigating the role of *HCP5* in CC cell proliferation, the cells were seeded in 96-well plates at 48 h after transfection; then, the MTT assay was performed as described above.

### 4.6. Cell Colony Formation Assay

CC cells were counted and seeded at 2000 cells per well in a six-well plate. Fourteen days after being continuously cultured, cell colonies were washed with PBS twice, fixed in formalin for 30 min, and stained with 0.1% crystal violet for another 20 min. Then, the cell colonies were imaged and counted.

### 4.7. Wound-Healing Assays 

The cells were cultured in a 24-well plate in the wound-healing assay. Post different treatments, the cell monolayer was scratched with a 200 µL pipette tip. The microscope (Olympus, Tokyo, Japan) took representative images of the scratch at 0 h and 48 h. The percentage of wound closure was measured via the ImageJ 1.52p software. 

### 4.8. Real-Time Cellular Analysis (RTCA)

The migratory ability of CC cells was detected using xCELLigence RTCA (ACEA Biosciences, San Diego, CA, USA). Briefly, post-transfected CC cells were resuspended in a serum-free medium, and 10,000 CC cells were added per upper chamber. Meanwhile, the 165 µL medium with 10% FBS was added to the lower section. Then, the cell plate was incubated at 37 °C and 5% CO_2_, and the cell index was detected uninterrupted using the RTCA software 2020R1 until the end of the experiment. 

### 4.9. Cell Apoptosis Assays 

The cell apoptosis detection was performed using the FITC/Annexin V Apoptosis Detection Kit (BD, 556547, Franklin Lakes, NJ, USA). Briefly, the cells were harvested post-treatment, followed by the addition of 5 µL Annexin V-FITC and 5 µL propidum iodide into the cell suspension. Then, the cell apoptosis ratio was analyzed via flow cytometry (Beckman, Brea, CA, USA).

### 4.10. Western Blot Assays 

The whole proteins from CC cells or xenograft tissues were extracted using the RIPA lysis buffer (Lablead, Beijing, China), and then western blotting was conducted as described previously [41]. The following antibodies were used: p-STAT3 (1:1000, CST, Worcester, MA, USA), STAT3 (1:1000, CST, USA), p65 (1:1000, CST, USA), Lamin B1 (1:1000, CST, USA), DDIT4 (1:1000, Proteintech, San Diego, CA, USA), β-Tubblin (1:1000, Transgene Biotech, Shanghai, China), and β-actin (1:1000, Transgen Biotech, China). 

### 4.11. Luciferase Reporter Assay

To explore the transcription regulation of p65 on *HCP5*, wild-type or mutated *HCP5* promoter sequences were cloned into a pGL4.10 promoter reporter vector (Promega, Fitchburg, WI, USA) to generate a pGL4.1/HCP5-WT vector and a pGL4.1/HCP5-MUT vector. Then, the p65 overexpression plasmid and these vectors were co-transfected into CC cells using Lipofectamine 3000. 

As for elucidating the ceRNA mechanism among *HCP5*, *miR-139-5p*, and DDIT4, the wild-type or mutant *HCP5* fragment and the 3′untranslated region (UTR) of DDIT4 were inserted into the pmirREPORTER luciferase reporter vector (Promega, USA). CC cells were individually transfected with HCP5-WT, HCP5-MUT, DDIT4-WT, or DDIT4-MUT vectors, together with *miR-139-5p* mimics. Forty-eight hours after transfection, the luciferase activities were determined using the Dual-Luciferase Assay Kit (Promega, USA), according to the manufacturer’s instructions. Renilla luciferase activities were set as the standardized control. 

### 4.12. RIP Assay

The RIP assay was conducted using a Magna RIP Kit (Millipore, Burlington, MA, USA), according to the manufacturer’s instructions. Briefly, the lysates of CC cells were incubated overnight with the beads coated with the anti-Ago2 antibody or the control IgG (Proteintech, CA, USA) antibody at 4 °C under rotation. Subsequently, the precipitated RNA was purified and analyzed via qRT-PCR.

### 4.13. ChIP Assay

The EZ-Magna CHIP kit (Millipore, MA, USA) was used to perform the ChIP assay, followed by the previously published methods [42]. The DNA fragments precipitated by the p65 (CST, Worcester, MA, USA) or IgG (Proteintech, San Diego, CA, USA) antibodies were purified and detected using qRT-PCR. 

### 4.14. Animal Experiments

TGR5 KO female mice (on C57BL/6J background; Merck Research Laboratories, Kenilworth, NJ) were raised in a pathogen-free animal facility. Eight-week-old wild-type (WT) (C57BL/6J), and four-week-old female BALB/c mice were purchased from Beijing Experimental Animal Center. All animal experiments were carried out in compliance with the NIH Guide for the Care and Use of Laboratory Animals. 

For LPS-induced cervical inflammation, WT and TGR5 KO mice fasted overnight, then were injected intraperitoneally with LPS (20 mg/kg body weight) or PBS. At 6 h post-treatment, mice were sacrificed, and the cervical tissues were collected for further investigation. 

For the study of the roles of TGR5 activation in tumorigenesis in vivo, nude mice were subcutaneously injected with HeLa cells at the right axilla (3 × 10^6^ cells per mouse). Once the average volume of tumors reached about 100 mm^3^, the mice were randomized into two groups and received intraperitoneal injections of BAR501 (20 mg/kg body weight) or the vehicle every two days. The length and width of the xenograft tumor were measured during treatment. Once treatment was finished, the mice were humanely executed, and the tumors were collected and weighed. Tumor volumes were calculated using the formula: ½ × (length × width^2^).

For investigating the function of *HCP5* in tumor growth in vivo, HeLa cells (3 × 10^6^ cells per mouse) transfected with sh-NC and sh-HCP5 were suspended in 150 µL PBS and subsequently injected into the flank of nude mice. The tumor volume was measured every two days during the experimental period. Three weeks after injection, mice were sacrificed, and the tumors were removed and weighed. 

### 4.15. Quantification and Statistical Analysis

All results are shown as the mean ± standard deviation and include at least three independent experiments unless stated otherwise. A *p*-value less than 0.05 was significant. The GraphPad Prism 8 software was utilized for all statistical evaluations. For the assessment of differences between two groups, unpaired two-tailed Student’s *t*-tests were employed, while one-way or two-way ANOVA was applied for the examination of differences across more than two groups, followed by Tukey’s multiple comparison test.

## 5. Conclusions

In conclusion, this study reveals that TGR5 suppresses the development of CC by regulating the *HCP5*/*miR-139-5p*/*DDIT4* axis, suggesting that handling the novel TGR5/*HCP5*/*miR-139-5p*/*DDIT4* pathway could be a potential strategy for CC treatment.

## Data Availability

The original contributions presented in the study are included in the article and Supplementary Materials. Further inquiries can be directed to the corresponding authors.

## Supplementary Materials

The following supporting information can be downloaded at: https://www.mdpi.com/article/10.3390/ijms25168932/s1.
