# Supplementary material for: Activation of the G Protein-Coupled Bile Acid Receptor TGR5 Modulates the HCP5/miR-139-5p/DDIT4 Axis to Antagonize Cervical Cancer Progression
Source: Int J Mol Sci. 2024 Aug 16;25(16):8932. doi: 10.3390/ijms25168932 (PMC11354701; doi:10.3390/ijms25168932)
Supplement: Supplementary file 1 [file ijms-25-08932-s001.zip › ijms-3123736-supplementary.pdf]

Table S1. Sequences of siRNA, shRNA and primers.

Types	Name	Sequences 5'-3'
Overexpression Plasmid	HCP5-oe-F	GGGGCTAGCGACTCAGATTCTCCCCAGAG
	HCP5-oe-R	GGGGAATTCTTCATGTGGGATCCACAAC
	DDIT4-oe-F	GGGGCTAGCGCCACCATGCCTAGCCTTTGGGACCG
	DDIT4-oe-R	GGGGAATTCTCAACACTCCTCAATGAGCAGCT
	p65-oe-F	CCCAAGCTT ATGGACGAACTGTTCCCCCT
	p65-oe-R	CTAGTCTAGATTAGGAGCTGATCTGACTCAGCA
shRNA	sh-HCP5-1	GGTCTGGGCTCTTGGAAATCA
	sh-HCP5-2	GGACGATTCTCCTCACACTTA
siRNA	si-DDIT4	
Luciferase Plasmids	HCP5 promoter-F	CGGGGTACCACGGTGGTTCACGCCTGTAA
	HCP5 promoter-R	CCCAAGCTTGCGCGGTCGCTTACATAACC
	HCP5-WT-F	GGGGAGCTCCCAGGACCTTCTACCCTGCAG
	HCP5-WT -R	GGGTCTAGAAGCTTGCAGGAAGCACTCTG
	DDIT4-WT-F	CCCAAGCTTAACTGAGGCAGCCACCTAAGG
	DDIT4-WT-R	AAAGAGCTCAGCTGCTCTCTGGATGTCACAC

Table S2. Sequences of qRT-PCR primers.

Species	Gene	Forward primer5'-3'	Reverse primer5'-3'
Human	β-actin	AAGATCAAGATCATTGCTCCTCCTG	GCAACTAAGTCATAGTCCGCCT
	HCP5	TCCAGCCACTATTGGCCATC	TGCAGAGGCCCTACTTCTCT
	HCP5（ChIP）	GGTTCACGCCTGTAATCCC	ACCACCAAGCCCGTCTAA
	DDIT4	TGACCTGTGAGGGGTGTAAA	GTCGACACTCTTGACACTTT
	U6	CTCGCTTCGGCAGCACA	AACGCTTCACGAATTTGCGT
	miR-139-5p RT	CTCAACTGGTGTCGTGGAGTCGGCAATTCAGTTGAGACTGGAG	
	miR-139-5p	ACACTCCAGCTGGGTCTACAGTGCACGTGTCTC	TGGTGTCGTGGAGTCG
Mouse	36b4	GCTGTTGGCCAATAAGGTGC	ATGCCCAAAGCCTGGAAGAA
	ICAM1	TGTGACCAGCCCAAGTTGTT	TGGAGTCCAGTACACGGTGA
	C3	TGTGGAGTTCGAGGAACCCT	AGTCCGGATCTAGGCAGGTT
	IP-10	TGCCTCATCCTGCTGGGTCT	CCCTGCGAGCCTATCCTGCC
	MCP-1	ATGCTTCTGGGCCTGCTGTT	CAGCTTCTTTGGGACACCTG
	MMP7	AGCACTGCATGCTTTCTAATTATCT	CAACATCTGGCTCCACAC
	MMP9	TCATGGTCCACCTTGTTCAC	AAGTCTCAGAAGGTGGATCC
	MMP12	TGATGGCAAAGGTGGTACAC	CCAAGGAATGGCCAAGTTCA

Table S3. Significant lncRNAs after filtration in our own RNA sequencing data

Gene	gene_type	Position	Strand	log2FC	p-value
PCF11-AS1	lncRNA	chr11:82896563-82898526	-	-6.77844223	0.017344062
LINC00858	lncRNA	chr10:86039736-86054415	+	-6.624490865	0.022579451
LINC01126	lncRNA	chr2:43454350-43455994	+	-6.361456459	0.034098574
MIR210HG	lncRNA	chr11:565657-568457	-	-6.353636955	0.034489558
RUSC1-AS1	lncRNA	chr1:155286645-155293967	-	-5.594946589	0.001770426

LINC00494	lncRNA	chr20:46988654-46999381	+	-5.434937057	0.02664489
SMC5-AS1	lncRNA	chr9:72830975-72873790	-	-5.187627003	0.040192942
TMPO-AS1	lncRNA	chr12:98906751-98910004	-	-4.929610672	0.017052647
LHX4-AS1	lncRNA	chr1:180238798-180243816	-	-4.781359714	0.018506497
NFYC-AS1	lncRNA	chr1:41154752-41157933	-	-4.744958385	0.03191603
LINC01224	lncRNA	chr19:23582035-23598876	-	-4.222392421	0.040503577
LOC401261	lncRNA	chr6:42751234-42759061	+	-4.192895524	0.012685565
LOC100507437	lncRNA	chr14:105883918-105886076	-	-4.060641351	0.04787253
RAB30-AS1	lncRNA	chr11:82783108-82784754	+	-3.90902634	0.04173517
LINC01004	lncRNA	chr7:104622194-104631612	-	-3.53447273	0.005937211
CTBP1-AS2	lncRNA	chr4:1243228-1246795	+	-3.459237303	0.008664564
LOC108783654	lncRNA	chr17:40704454-40706820	-	-3.321928095	0.017894914
IGFL2-AS1	lncRNA	chr19:46692286-46706319	-	-3.209808438	0.003347308
LINC00641	lncRNA	chr14:21668238-21675059	-	-2.903130939	0.017930445
**HCP5**	lncRNA	chr6:31430957-31433586	+	-2.885179598	0.01548361
MIR22HG	lncRNA	chr17:1614798-1619566	-	-2.882369506	0.018799763
KMT2E-AS1	lncRNA	chr7:104650989-104654588	-	-2.602664502	0.048665936
LINC00659	lncRNA	chr20:61405473-61408208	-	2.583569265	0.049828518
NPTN-IT1	lncRNA	chr15:73859279-73861884	-	2.584426482	0.024396979
LOC284454	lncRNA	chr19:13945330-13947103	-	2.590911767	0.025703833
LINC01578	lncRNA	chr15:93426073-93441977	+	2.686251555	0.014902803
TPT1-AS1	lncRNA	chr13:45915480-45965618	+	3.557413315	0.004701826
LINC00260	lncRNA	chr1:203699705-203700981	+	3.570240995	0.005432699
H19	lncRNA	chr11:2016406-2022696	-	3.865971941	0.000795934
LINC00887	lncRNA	chr3:194018988-194030593	-	4.01346226	0.019980199
LINC01564	lncRNA	chr6:53493178-53496192	+	5.221587121	0.013073156

ITGB2-AS1	lncRNA	chr21:46340950-46349595	+	5.439385676	0.005324354
LOC100130872	lncRNA	chr4:1189571-1202750	-	6.408329741	0.001246787
FLJ22447	lncRNA	chr14:62037258-62121431	+	7.112439507	0.007478529
LINC01300	lncRNA	chr8:142350648-142354720	+	7.192292814	0.022902921
ENSG00000269473.1	lincRNA	chr19:58951815-58957216	+	-6.222392421	0.041438218
ENSG00000269564.1	lincRNA	chr19:54292036-54292422	+	-6.106199404	0.048159478
ENSG00000255121.2	lincRNA	chr11:118865534-118868714	-	-5.650253961	0.034685894
ENSG00000273329.1	lincRNA	chr7:129244389-129251471	-	-4.476813697	0.00547749
ENSG00000269416.1	lincRNA	chr19:23582272-23598876	-	-4.415037499	0.02357738
ENSG00000237491.4	lincRNA	chr1:714150-745440	+	-4.333539385	0.028164251
ENSG00000268621.1	lincRNA	chr19:46699035-46706340	-	-4.325070734	0.000180755
ENSG00000270012.1	lincRNA	chrX:49129517-49132231	+	-4.222392421	0.030905556
ENSG00000270081.1	lincRNA	chr2:128601127-128603261	-	-3.877038478	0.036927972
ENSG00000240476.1	lincRNA	chr3:98699902-98701940	+	2.729442486	0.032700935
ENSG00000224959.1	lincRNA	chr2:112249520-112252388	-	3.186413124	0.008535686
ENSG00000261087.1	lincRNA	chr8:102179033-102181857	-	3.31624339	0.03487007
ENSG00000261068.1	lincRNA	chr6:42059976-42061997	-	3.627131955	0.005282736
ENSG00000269967.1	lincRNA	chr1:32317514-32387442	-	3.890542602	0.011700566
ENSG00000232324.1	lincRNA	chr19:54368017-54369394	+	4.87036472	0.026805602
NPPA-AS1	antisense_RNA	chr1:11900376-11907673	+	2.451988635	0.041680402
TALAM1	antisense_RNA	chr11:65266783-65274903	-	4.833589672	6.36E-05
ENSG00000266680.1	antisense	chr6:64280910-64282313	-	-4.473931188	0.024469615
ENSG00000270504.1	antisense	chr6:3751345-3754105	+	-2.384376947	0.046979035

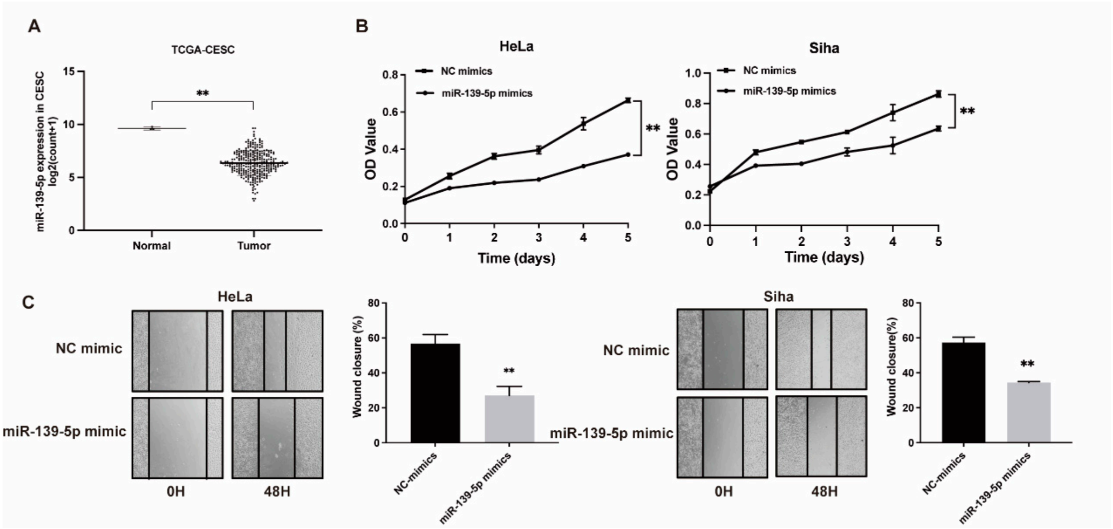

Figure S1. miR-139-5p attenuated the proliferation and migration of CC cells *in vitro*.

A. The expression of miR-139-5p in tumor tissues compared to normal tissues in TCGA-CESC.
B. The MTT assay in HeLa and Siha cells after miR-39-5p overexpression.
C. Wound healing assay was conducted to examine the migratory ability of CC cells transfected with negative mimics (NC mimics) or miR-139-5p mimics.

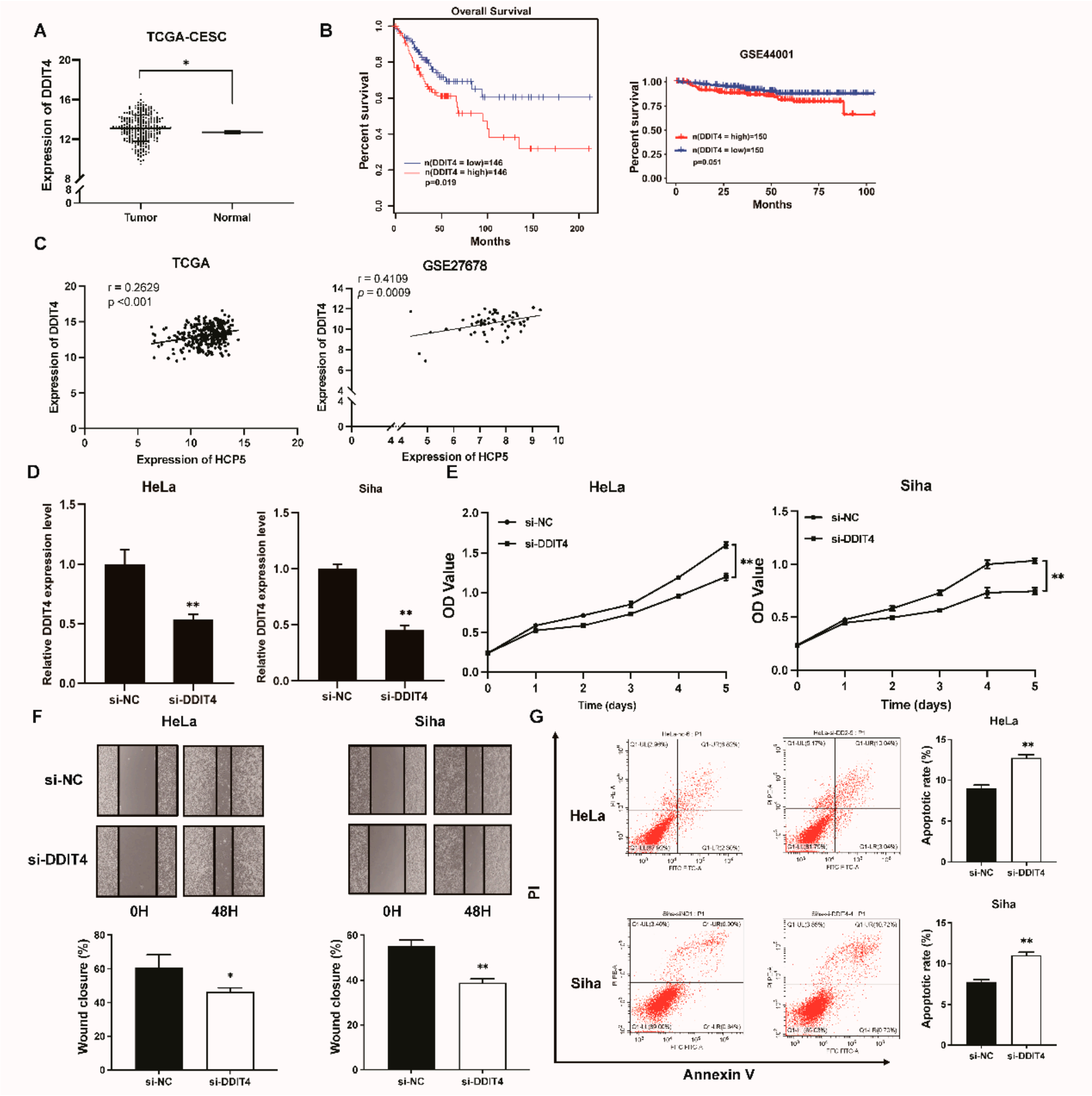

Figure S2. DDIT4 knockdown suppressed CC cell proliferation and migration while accelerating the cell apoptosis process.

A. The expression of DDIT4 in tumor tissues compared to normal tissues in TCGA-CESC.

B. Kaplan-Meier analysis was used to verify the prognosis of CC patients with high DDIT4 expression compared to low DDIT4 expression based on TCGA-CESC and GSE44001 data.

C. The correlation analysis of HCP5 and DDIT4 in cancer tissues based on TCGA-CESC and GSE27678.

D. The analysis of DDIT4 level in HeLa and Siha cell after transfected with si-NC and si-DDIT4 were conducted via qRT-PCR.

E, F. Cell proliferation and migration of CC cells post-silencing DDIT4 were verified through MTT assay and wound healing assay, respectively.

G. Flow cytometry was performed to detect the cell apoptosis in CC cells transfected with si-NC and si-DDIT4.
